# 3,5-Dinitro-*N*-(1,3-thia­zol-2-yl)benzamide monohydrate

**DOI:** 10.1107/S1600536811005228

**Published:** 2011-02-19

**Authors:** Sohail Saeed, Naghmana Rashid, Wing-Tak Wong, Rizwan Hussain

**Affiliations:** a Department of Chemistry, Research Complex, Allama Iqbal Open University, Islamabad 44000, Pakistan; b Department of Chemistry, The University of Hong Kong, Pokfulam Road, Pokfulam, Hong Kong SAR, People’s Republic of China; c National Engineering & Scientific Commission, PO Box 2801, Islamabad, Pakistan

## Abstract

In the title compound, C_10_H_6_N_4_O_5_S·H_2_O, the thia­zole ring is twisted at a dihedral angle of 25.87 (7)° with respect to the benzene ring. The water mol­ecule is linked with the benzamide mol­ecules *via* N—H⋯O, O—H⋯N and O—H⋯O hydrogen bonds. In the crystal, π–π stacking is observed between nearly parallel [dihedral angle = 7.02 (7)°] thia­zole and benzene rings of adjacent mol­ecules, the centroid–centroid distances being 3.7107 (9) and 3.7158 (9) Å, respectively.

## Related literature

For the effect of substituents on the structures of benzanilides, see: Gowda *et al.* (2008 ▶).
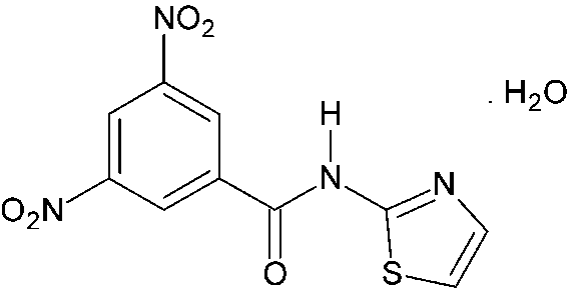


## Experimental

### 

#### Crystal data

C_10_H_6_N_4_O_5_S·H_2_O
*M* *_r_* = 312.26
Monoclinic, 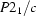

*a* = 13.7075 (12) Å
*b* = 6.9734 (6) Å
*c* = 13.8507 (13) Å
β = 108.512 (1)°
*V* = 1255.45 (19) Å^3^
*Z* = 4
Mo *K*α radiation
μ = 0.30 mm^−1^
*T* = 296 K
0.28 × 0.07 × 0.06 mm

#### Data collection

Bruker SMART 1000 CCD diffractometer
Absorption correction: multi-scan (*SADABS*; Sheldrick, 2004 ▶) *T*_min_ = 0.922, *T*_max_ = 0.983
6750 measured reflections
2214 independent reflections
1937 reflections with *I* > 2σ(*I*)
*R*_int_ = 0.015

#### Refinement

*R*[*F* ^2^ > 2σ(*F* ^2^)] = 0.029
*wR*(*F* ^2^) = 0.088
*S* = 1.02
2214 reflections
203 parameters
H atoms treated by a mixture of independent and constrained refinement
Δρ_max_ = 0.19 e Å^−3^
Δρ_min_ = −0.21 e Å^−3^

### 

Data collection: *SMART* (Bruker, 1998 ▶); cell refinement: *SAINT* (Bruker, 2006 ▶); data reduction: *SAINT* and *CrystalStructure* (Rigaku/MSC, 2006 ▶); program(s) used to solve structure: *SHELXS97* (Sheldrick, 2008 ▶); program(s) used to refine structure: *SHELXL97* (Sheldrick, 2008 ▶); molecular graphics: *ORTEPII* (Johnson, 1976 ▶); software used to prepare material for publication: *SHELXL97*.

## Supplementary Material

Crystal structure: contains datablocks global, I. DOI: 10.1107/S1600536811005228/xu5155sup1.cif

Structure factors: contains datablocks I. DOI: 10.1107/S1600536811005228/xu5155Isup2.hkl

Additional supplementary materials:  crystallographic information; 3D view; checkCIF report

## Figures and Tables

**Table 1:** Hydrogen-bond geometry (Å, °)

*D*—H⋯*A*	*D*—H	H⋯*A*	*D*⋯*A*	*D*—H⋯*A*
N2—H2*N*⋯O6	0.871 (19)	1.974 (19)	2.8313 (18)	167.9 (17)
O6—H6*B*⋯O1^i^	0.75 (3)	2.38 (2)	3.0350 (19)	147 (2)
O6—H6*C*⋯N1^ii^	0.84 (3)	2.14 (3)	2.964 (2)	168 (2)

Symmetry codes: (i) 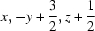; (ii) 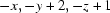.

**Fig. 1.**
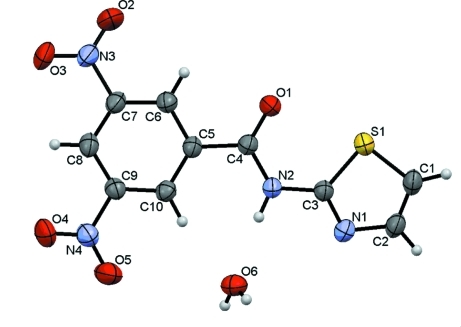
The ORTEP plot of the compound was shown at 50% probability thermal ellipsoids.

## supplementary crystallographic information

### Comment

In the present work, the structure of 3,5-dinitro-*N*-thiazol-2-yl-benzamide monohydrate has been determined to explore the effect of substituents on the structure of benzanilides (Gowda *et al.*, 2008).

The molecule is not planar. The thiazole ring is twisted to the benzene ring at a dihedral angle of 25.87 (7)°. The nitro groups are 12.30 (20)° and 15.68 (15)° from the phenyl ring plane of C5—C10. The thiazole ring is making a dihedral angle of 11.90 (2)° with the amido group which in turn makes a dihedral angle of 14.01 (4)° with the phenyl ring plane of C5—C10.

There are intermolecular N—H···O, O—H···N and O—H···O H-bond interactions, which link the molecules to form 2-D networks in the crystal lattice. There are also weak π···π interactions between neighbouring rings in the crystal lattice.

### Experimental

A solution of 3,5-dinitrobenzoyl chloride (0.01 mol) and 2-aminothiazole (0.01 mol) in anhydrous acetone was refluxed for 4 h. After completion of the reaction, the crude solid product was filtered, washed with water and purified by re-crystallization from ethyl acetate/water.

### Refinement

All of the C-bound H atoms are observable from difference Fourier map but are all placed at geometrical positions with C—H = 0.93 Å for phenyl H-atoms. All C-bound H-atoms are refined using riding model with *U*_iso_(H) = 1.2*U*_eq_(C). Both the N– and O-bound H-atoms were located from a difference Fourier map and refined isotropically.

### Crystal data

**Table:**

C_10_H_6_N_4_O_5_S·H_2_O	*F*(000) = 640
*M**_r_* = 312.26	*D*_x_ = 1.652 Mg m^−^^3^
Monoclinic, *P*2_1_/*c*	Mo *K*α radiation, λ = 0.71073 Å
Hall symbol: -P 2ybc	Cell parameters from 9023 reflections
*a* = 13.7075 (12) Å	θ = 1.8–25.0°
*b* = 6.9734 (6) Å	µ = 0.30 mm^−^^1^
*c* = 13.8507 (13) Å	*T* = 296 K
β = 108.512 (1)°	Needle, colourless
*V* = 1255.45 (19) Å^3^	0.28 × 0.07 × 0.06 mm
*Z* = 4	

### Data collection

**Table:**

Bruker SMART 1000 CCD diffractometer	2214 independent reflections
Radiation source: fine-focus sealed tube	1937 reflections with *I* > 2σ(*I*)
graphite	*R*_int_ = 0.015
ω scans	θ_max_ = 25.0°, θ_min_ = 3.0°
Absorption correction: multi-scan (*SADABS*; Sheldrick, 2004)	*h* = −16→15
*T*_min_ = 0.922, *T*_max_ = 0.983	*k* = −8→8
6750 measured reflections	*l* = −16→16

### Refinement

**Table:**

Refinement on *F*^2^	Secondary atom site location: difference Fourier map
Least-squares matrix: full	Hydrogen site location: inferred from neighbouring sites
*R*[*F*^2^ > 2σ(*F*^2^)] = 0.029	H atoms treated by a mixture of independent and constrained refinement
*wR*(*F*^2^) = 0.088	*w* = 1/[σ^2^(*F*_o_^2^) + (0.0569*P*)^2^ + 0.2612*P*] where *P* = (*F*_o_^2^ + 2*F*_c_^2^)/3
*S* = 1.02	(Δ/σ)_max_ = 0.001
2214 reflections	Δρ_max_ = 0.19 e Å^−^^3^
203 parameters	Δρ_min_ = −0.21 e Å^−^^3^
0 restraints	Extinction correction: *SHELXL97* (Sheldrick, 2008), Fc^*^=kFc[1+0.001xFc^2^λ^3^/sin(2θ)]^-1/4^
Primary atom site location: structure-invariant direct methods	Extinction coefficient: 0.0069 (14)

### Special details

**Table:**

Geometry. All e.s.d.'s (except the e.s.d. in the dihedral angle between two l.s. planes) are estimated using the full covariance matrix. The cell e.s.d.'s are taken into account individually in the estimation of e.s.d.'s in distances, angles and torsion angles; correlations between e.s.d.'s in cell parameters are only used when they are defined by crystal symmetry. An approximate (isotropic) treatment of cell e.s.d.'s is used for estimating e.s.d.'s involving l.s. planes.
Refinement. Refinement of *F*^2^ against ALL reflections. The weighted *R*-factor *wR* and goodness of fit *S* are based on *F*^2^, conventional *R*-factors *R* are based on *F*, with *F* set to zero for negative *F*^2^. The threshold expression of *F*^2^ > σ(*F*^2^) is used only for calculating *R*-factors(gt) *etc*. and is not relevant to the choice of reflections for refinement. *R*-factors based on *F*^2^ are statistically about twice as large as those based on *F*, and *R*- factors based on ALL data will be even larger.

### Fractional atomic coordinates and isotropic or equivalent isotropic displacement parameters (Å^2^)

**Table:**

	*x*	*y*	*z*	*U*_iso_*/*U*_eq_	
S1	−0.17423 (3)	0.90512 (6)	0.12088 (3)	0.03884 (16)	
O1	0.02312 (8)	0.95294 (19)	0.11971 (8)	0.0469 (3)	
O2	0.35736 (11)	1.1250 (3)	0.08520 (11)	0.0793 (5)	
O3	0.49514 (9)	1.0180 (3)	0.19517 (10)	0.0669 (4)	
O4	0.48076 (9)	0.7824 (2)	0.51780 (10)	0.0590 (4)	
O5	0.34262 (11)	0.8314 (3)	0.55577 (10)	0.0777 (5)	
N1	−0.14748 (10)	0.9651 (2)	0.31080 (10)	0.0403 (3)	
N2	0.00921 (9)	0.94048 (19)	0.27755 (10)	0.0351 (3)	
H2N	0.0335 (15)	0.929 (3)	0.3435 (15)	0.046 (5)*	
N3	0.40286 (11)	1.0453 (2)	0.16479 (11)	0.0505 (4)	
N4	0.39071 (10)	0.8282 (2)	0.49550 (10)	0.0453 (4)	
C1	−0.28028 (12)	0.9200 (2)	0.16129 (13)	0.0425 (4)	
H1	−0.3480	0.9069	0.1193	0.051*	
C2	−0.25176 (12)	0.9531 (2)	0.26157 (13)	0.0429 (4)	
H2	−0.2996	0.9672	0.2960	0.052*	
C3	−0.09807 (11)	0.9410 (2)	0.24567 (11)	0.0331 (3)	
C4	0.06477 (11)	0.9452 (2)	0.21168 (11)	0.0338 (3)	
C5	0.17976 (11)	0.9424 (2)	0.25696 (11)	0.0324 (3)	
C6	0.23598 (11)	0.9885 (2)	0.19236 (12)	0.0368 (4)	
H6	0.2027	1.0230	0.1251	0.044*	
C7	0.34216 (11)	0.9820 (2)	0.23006 (12)	0.0379 (4)	
C8	0.39520 (12)	0.9255 (2)	0.32777 (12)	0.0382 (4)	
H8	0.4665	0.9150	0.3507	0.046*	
C9	0.33678 (11)	0.8856 (2)	0.38975 (11)	0.0352 (3)	
C10	0.23081 (11)	0.8948 (2)	0.35798 (11)	0.0340 (3)	
H10	0.1944	0.8698	0.4029	0.041*	
O6	0.07962 (11)	0.8457 (2)	0.48713 (10)	0.0503 (3)	
H6B	0.0571 (18)	0.752 (4)	0.4970 (18)	0.076 (9)*	
H6C	0.0932 (18)	0.912 (3)	0.5399 (19)	0.073 (8)*	

### Atomic displacement parameters (Å^2^)

**Table:**

	*U*^11^	*U*^22^	*U*^33^	*U*^12^	*U*^13^	*U*^23^
S1	0.0299 (2)	0.0482 (3)	0.0354 (2)	−0.00058 (15)	0.00610 (16)	0.00073 (16)
O1	0.0341 (6)	0.0737 (9)	0.0322 (6)	0.0033 (5)	0.0092 (5)	0.0028 (5)
O2	0.0531 (8)	0.1362 (15)	0.0526 (8)	−0.0101 (8)	0.0222 (7)	0.0276 (9)
O3	0.0322 (7)	0.1079 (12)	0.0660 (9)	−0.0099 (7)	0.0233 (6)	−0.0043 (8)
O4	0.0356 (6)	0.0801 (10)	0.0533 (7)	0.0133 (6)	0.0030 (5)	0.0077 (7)
O5	0.0573 (9)	0.1376 (15)	0.0406 (7)	0.0245 (9)	0.0190 (6)	0.0190 (8)
N1	0.0314 (7)	0.0498 (8)	0.0414 (7)	0.0005 (6)	0.0139 (6)	0.0001 (6)
N2	0.0260 (7)	0.0474 (8)	0.0312 (7)	−0.0003 (5)	0.0078 (5)	−0.0010 (6)
N3	0.0379 (8)	0.0734 (11)	0.0447 (8)	−0.0118 (7)	0.0197 (7)	−0.0075 (7)
N4	0.0388 (8)	0.0546 (9)	0.0393 (7)	0.0046 (6)	0.0076 (6)	0.0011 (6)
C1	0.0275 (8)	0.0450 (9)	0.0529 (10)	−0.0009 (6)	0.0096 (7)	0.0055 (7)
C2	0.0289 (8)	0.0485 (10)	0.0540 (10)	0.0014 (6)	0.0170 (7)	0.0057 (8)
C3	0.0285 (7)	0.0355 (8)	0.0349 (8)	0.0006 (6)	0.0097 (6)	0.0016 (6)
C4	0.0299 (8)	0.0377 (8)	0.0340 (8)	0.0001 (6)	0.0107 (6)	−0.0008 (6)
C5	0.0280 (8)	0.0339 (8)	0.0355 (8)	−0.0005 (5)	0.0106 (6)	−0.0036 (6)
C6	0.0329 (8)	0.0437 (9)	0.0339 (8)	−0.0024 (6)	0.0107 (6)	−0.0032 (7)
C7	0.0329 (8)	0.0449 (9)	0.0398 (8)	−0.0054 (6)	0.0169 (7)	−0.0069 (7)
C8	0.0281 (7)	0.0435 (9)	0.0424 (9)	−0.0005 (6)	0.0105 (6)	−0.0091 (7)
C9	0.0322 (8)	0.0377 (8)	0.0341 (8)	0.0024 (6)	0.0082 (6)	−0.0037 (6)
C10	0.0330 (8)	0.0358 (8)	0.0352 (8)	0.0008 (6)	0.0137 (6)	−0.0021 (6)
O6	0.0545 (8)	0.0628 (9)	0.0371 (7)	−0.0040 (7)	0.0195 (6)	−0.0006 (6)

### Geometric parameters (Å, °)

**Table:**

S1—C1	1.7187 (16)	C1—H1	0.9300
S1—C3	1.7304 (15)	C2—H2	0.9300
O1—C4	1.2205 (18)	C4—C5	1.500 (2)
O2—N3	1.215 (2)	C5—C10	1.391 (2)
O3—N3	1.2147 (18)	C5—C6	1.391 (2)
O4—N4	1.2162 (17)	C6—C7	1.382 (2)
O5—N4	1.2171 (18)	C6—H6	0.9300
N1—C3	1.299 (2)	C7—C8	1.375 (2)
N1—C2	1.377 (2)	C8—C9	1.376 (2)
N2—C4	1.3618 (19)	C8—H8	0.9300
N2—C3	1.3947 (19)	C9—C10	1.379 (2)
N2—H2N	0.871 (19)	C10—H10	0.9300
N3—C7	1.477 (2)	O6—H6B	0.75 (3)
N4—C9	1.471 (2)	O6—H6C	0.84 (3)
C1—C2	1.338 (2)		
			
C1—S1—C3	88.30 (8)	O1—C4—C5	121.21 (13)
C3—N1—C2	109.65 (14)	N2—C4—C5	117.15 (13)
C4—N2—C3	123.06 (13)	C10—C5—C6	119.83 (13)
C4—N2—H2N	126.6 (12)	C10—C5—C4	123.35 (13)
C3—N2—H2N	110.2 (12)	C6—C5—C4	116.81 (13)
O3—N3—O2	124.34 (15)	C7—C6—C5	118.77 (14)
O3—N3—C7	117.90 (15)	C7—C6—H6	120.6
O2—N3—C7	117.74 (14)	C5—C6—H6	120.6
O4—N4—O5	123.90 (14)	C8—C7—C6	123.06 (14)
O4—N4—C9	118.23 (13)	C8—C7—N3	117.61 (14)
O5—N4—C9	117.87 (13)	C6—C7—N3	119.28 (14)
C2—C1—S1	110.49 (12)	C7—C8—C9	116.21 (14)
C2—C1—H1	124.8	C7—C8—H8	121.9
S1—C1—H1	124.8	C9—C8—H8	121.9
C1—C2—N1	116.08 (14)	C8—C9—C10	123.66 (14)
C1—C2—H2	122.0	C8—C9—N4	117.92 (14)
N1—C2—H2	122.0	C10—C9—N4	118.41 (13)
N1—C3—N2	120.67 (14)	C9—C10—C5	118.33 (13)
N1—C3—S1	115.47 (11)	C9—C10—H10	120.8
N2—C3—S1	123.84 (11)	C5—C10—H10	120.8
O1—C4—N2	121.63 (13)	H6B—O6—H6C	108 (2)

### Hydrogen-bond geometry (Å, °)

**Table:**

*D*—H···*A*	*D*—H	H···*A*	*D*···*A*	*D*—H···*A*
N2—H2N···O6	0.871 (19)	1.974 (19)	2.8313 (18)	167.9 (17)
O6—H6B···O1^i^	0.75 (3)	2.38 (2)	3.0350 (19)	147 (2)
O6—H6C···N1^ii^	0.84 (3)	2.14 (3)	2.964 (2)	168 (2)

Symmetry codes: (i) *x*, −*y*+3/2, *z*+1/2; (ii) −*x*, −*y*+2, −*z*+1.

### Table 2 Table 1. π···π interactions (Å, °) *Cg1* and *Cg2* are centroids of the rings S1/N1/C1-C3 and C5-C10 respectively, *CgI···CgJ* is the distance between ring centroids. The dihedral angle is that between the planes of the rings *I* and *J*. *CgI*_Perp is the perpendicular distance of *CgI* from ring *J*. *CgJ*_Perp is the perpendicular distance of *CgJ* from ring *I*.

**Table:**

*I*	*J*	*CgI···CgJ*	Dihedral angle	*CgI*_Perp	*CgJ*_Perp
1	2^i^	3.7158 (9)	7.02 (7)	3.3718 (6)	-3.4374 (6)
1	2^ii^	3.7107 (9)	7.02 (7)	-3.3175 (6)	3.4409 (6)

symmetry operators: ^i^: -X,-1/2+Y,1/2-Z ^ii^: -X,1/2+Y,1/2-Z
